# Recreational physical activity and risk of triple negative breast cancer in the California Teachers Study

**DOI:** 10.1186/s13058-016-0723-3

**Published:** 2016-06-17

**Authors:** Huiyan Ma, Xinxin Xu, Jessica Clague, Yani Lu, Kayo Togawa, Sophia S. Wang, Christina A. Clarke, Eunjung Lee, Hannah L. Park, Jane Sullivan-Halley, Susan L. Neuhausen, Leslie Bernstein

**Affiliations:** Department of Population Sciences, Beckman Research Institute of City of Hope, 1500 East Duarte Road, Duarte, CA 91010 USA; Section of Environment and Radiation, International Agency for Research on Cancer, 150 Cours Albert Thomas, 69372 Lyon, France; Cancer Prevention Institute of California, 2201 Walnut Avenue, Fremont, CA 94538 USA; Department of Preventive Medicine, Keck School of Medicine, University of Southern California, 2001 N Soto Street, Los Angeles, CA 90032 USA; Department of Epidemiology, School of Medicine, University of California, Irvine, 224 Irvine Hall, Irvine, CA 92697 USA

**Keywords:** Physical activity, Breast cancer, Triple negative breast cancer, Luminal, Estrogen receptor, Progesterone receptor, HER2, Risk factors, BMI, Menopausal hormone therapy

## Abstract

**Background:**

Evidence has accumulated showing that recreational physical activity reduces breast cancer risk. However, it is unclear whether risk reduction pertains to specific receptor-defined subtypes. Moreover, few studies have examined whether changes in the amount of recreational physical activity during adulthood influence breast cancer risk.

**Methods:**

A total of 108,907 women, ages 22 to 79 years with no history of breast cancer when joining the California Teachers Study in 1995–1996, completed a baseline questionnaire and were eligible for the study. Through 2012, 5882 women were diagnosed with invasive breast cancer. Breast cancer subtypes were defined by the expression status of estrogen receptor (ER), progesterone receptor (PR), and human epidermal growth factor receptor 2 (HER2). Multivariable Cox proportional hazards models provided adjusted hazard ratios (HRs) and 95 % confidence intervals (CIs) for breast cancer overall and ER/PR/HER2-defined subtypes associated with long-term (from high school through age 54 or age at cohort entry, whichever was younger) and baseline (during 3 years prior to baseline) recreational physical activity. Among women who also completed a follow-up questionnaire at 10 years after baseline in 2005–2008 (54,686 women, 1406 with invasive breast cancer), risk associated with changes in the amount of recreational physical activity from baseline to the 10-year follow-up (during 3 years prior to the 10-year follow-up) was determined.

**Results:**

Both long-term and baseline strenuous recreational physical activity were inversely associated with risk of invasive breast cancer (*P*_trend_ ≤0.03). The observed associations were mainly confined to women with triple negative breast cancer (TNBC, ER–/PR–/HER2–, *P*_trend_ ≤0.02) or luminal A-like subtype (ER+ or PR+ plus HER2–) who were former users of menopausal hormone therapy at baseline (*P*_trend_ = 0.02, *P*_homogeneity of trends_ ≤0.03). Moreover, women who consistently engaged in the highest level (≥3.51 h/wk/y) of strenuous recreational physical activity between baseline and 10-year follow-up had the lowest risk of breast cancer (HR = 0.71, 95 % CI = 0.52–0.98) when compared to those who were consistently low (≤0.50 h/wk/y).

**Conclusions:**

Strenuous recreational physical activity is associated with lower breast cancer risk, especially TNBC. The benefit may be maximized by consistently engaging in high-intensity recreational physical activity during adulthood.

**Electronic supplementary material:**

The online version of this article (doi:10.1186/s13058-016-0723-3) contains supplementary material, which is available to authorized users.

## Background

Breast cancer is the most common cancer among American women, except for skin cancer; and it is the second leading cause of cancer death among American women, only exceeded by lung cancer [1]. Recreational physical activity may be one of the few universally available modifiable, protective factors for breast cancer. Previous epidemiologic studies showed that the most active women had, on average, a 25–30 % lower breast cancer risk than women in the lowest category of recreational physical activity [2]. However, it is unclear whether increased recreational physical activity protects against the development of all breast cancers or only certain breast cancer subtypes as defined by the expression status of the estrogen receptor (ER), progesterone receptor (PR), and human epidermal growth factor receptor 2 (HER2).

In the California Teachers Study (CTS), which has actively followed female California public school professionals for incidence of cancer and other outcomes since 1995, we previously reported that increased strenuous recreational physical activity, but not moderate intensity recreational physical activity, was associated with reduced risk of invasive breast cancer; moreover, the association was confined to ER-negative (ER–) as well as ER–/PR– invasive breast cancer in the analyses for ER-defined or ER/PR-defined subtypes [3]. That publication was based on the follow-up data of 110,599 women aged 79 years or younger who had no history of breast cancer at cohort entry from baseline through December 2002 (hereafter referred to as “7-year follow-up”). We were unable to evaluate risk of triple negative breast cancer (TNBC, ER–/PR–/HER2–) in the earlier publication because HER2 data were not available for cases diagnosed before 1999. To our knowledge, only the Women’s Health Initiative Cohort Study [4] and two case-control studies [5, 6] have investigated the association between recreational physical activity and TNBC risk.

We therefore assess risk of invasive breast cancer overall and by ER/PR/HER2-defined subtypes associated with long-term and baseline total (strenuous plus moderate intensity), strenuous intensity, and moderate intensity recreational physical activity using CTS data from baseline through December, 2012 (hereafter referred to as “17-year follow-up”). Furthermore, we assessed whether any associations of recreational physical activity with luminal A-like (ER+ or PR+ plus HER2–) or TNBC, two common subtypes, are modified by body mass index (BMI) and several aspects of menopausal hormone therapy (MHT) use at baseline (overall use, type of MHT used, duration of MHT use, or the cessation of MHT use). Moreover, we investigated whether breast cancer risk is influenced by changes in the amount of recreational physical activity reported at baseline and at the 10-year follow-up.

## Methods

### Study population

Details of the CTS have been described previously [7]. Briefly, the CTS is a prospective cohort study of 133,479 female public school professionals who were current, recent, or retired school employees and members of the California State Teachers Retirement System at the time the study began in 1995. Women joined the cohort by completing and mailing a baseline questionnaire in 1995–1996.

The potentially eligible participants for this analysis were 110,599 women aged 79 years or younger with no history of breast cancer at baseline studied in our previous analysis of the association between recreational physical activity and the risk of invasive breast cancer using 7-year follow-up data [3]. Based on additional information collected after the publication of that analysis, we excluded 41 women who had unknown breast cancer history, seven women who had history of invasive breast cancer prior to cohort entry according to updated data, and one woman who withdrew from the study in 2012. We also excluded women in the “unknown” categories when less than 0.7 % of the participants comprised the category, including 714 with unknown pregnancy, 379 with unknown breast biopsy history, and 550 with unknown smoking history. A total of 108,907 women remained in the analytic cohort.

Person-time of follow-up began with the date a participant completed her baseline questionnaire and ended with the first of the following events: a breast cancer diagnosis (in situ breast cancer: *n* = 1333, invasive breast cancer: *n* = 5882), relocation outside of California for more than 4 months (*n* = 11,645), death (*n* = 10,855), or end of follow-up (December 31, 2012, *n* = 79,192). New diagnoses of first primary breast cancer among cohort members after baseline through the end of follow-up were identified through annual linkages with the California Cancer Registry (CCR), a legally mandated statewide population-based cancer reporting system, which was modeled after the National Cancer Institute’s (NCI) Surveillance, Epidemiology, and End Results (SEER) registry program. The CCR is composed of three SEER registries and maintains the highest standards for data quality and completeness [8]. The NCI-designated SEER registries, including the three registries comprising the CCR, have routinely abstracted ER and PR status of breast cancers from medical records since 1990. The additional recording of HER2 status in California began in 1999 [9]. Among women diagnosed with invasive breast cancer during the follow-up, 1055 were diagnosed prior to 1999; 4827 women were diagnosed in 1999 or thereafter. According to previously published biomarker subtypes based on ER/PR/HER2 expression status [5, 10, 11], 4827 women were classified as having TNBC (ER–/PR–/HER2–, *n* = 348), luminal A-like (ER+ or PR+ plus HER2–, *n* = 2701), luminal B-like (ER+ or PR+ plus HER2+, *n* = 393), HER2-enriched (ER–/PR–/HER2+, *n* = 159), or unknown subtype primarily due to missing information on HER2 status (*n* = 1226).

For the analysis of the association between breast cancer risk and changes in the amount of recreational physical activity between baseline and 10-year follow-up (2005–2008), potentially eligible participants were 62,187 women who returned the 10-year follow-up questionnaire. Among those, we excluded women who moved out of California for more than 4 months (*n* = 4386) or were diagnosed with breast cancer (*n* = 2959) between baseline and completion of the 10-year follow-up questionnaire. We further excluded 156 women who did not update the information on recreational physical activity in their follow-up questionnaire, resulting in a total of 54,686 study participants, of whom 1406 were diagnosed with invasive breast cancer after submitting the 10-year follow-up questionnaire and before the end of follow-up.

### Data collection

Details regarding the measures of self-reported recreational physical activity have been published elsewhere [3]. Briefly, participants provided information at baseline on recreational physical activity for two intensity levels, strenuous and moderate, at each of six time periods (during high school, in age categories of 18–24, 25–34, 35–44 and 45–54 years, and recent physical activity during the 3 years prior to baseline, which is referred to as baseline recreational physical activity in this article). The recent physical activity was updated in the 10-year follow-up questionnaire (referred to as 10-year follow-up recreational physical activity in this article). Examples of strenuous exercise included running, jogging, swimming laps, racquetball, aerobics, calisthenics, and cycling on hills. Examples of moderate intensity exercise included brisk walking, golf, softball, volleyball, recreational tennis, and cycling on flat surfaces. For each intensity level and time period, participants reported the average number of hours per week (categories: none, 0.5, 1, 1.5, 2, 3, 4–6, 7–10, and ≥11) and the average number of months per year (categories: 1–3, 4–5, 7–9, and 10–12) that they engaged in such activities. For each intensity level and time period, we calculated average annual hours per week of activity by multiplying the hours per week by the portion of the year in which the woman engaged in the activity and dividing by 12 months. Within each time period, this value was assigned to each year of age comprising that time period. We assigned the midpoint value of the categories for average hours per week in making these calculations, and a value of 12 for the category 11 h/wk or more. In this way we calculated the average hours per week for each year of age from high school through age 54 years or through the age at cohort entry if the woman was less than 54 years of age at baseline. We then summed across all eligible years and calculated the average annual hours per week of long-term (activities from high school through age 54 or the participant’s age at cohort entry if younger than 54), baseline, and 10-year follow-up recreational physical activity for each intensity level. Furthermore, we summed long-term strenuous and long-term moderate activity to obtain total long-term recreational physical activity. When examined as predictors, hours per week of total recreational physical activity were categorized as 0–0.50, 0.51–2.50, 2.51–4.50, 4.51–7.00, and ≥7.01 h/wk/y; the categories used for strenuous intensity activity and for moderate intensity activity were 0–0.50, 0.51–2.00, 2.01–3.50, 3.51–5.00, and ≥5.01 h/wk/y.

The baseline questionnaire also collected detailed information on self-reported menopausal status and use of MHT. In this analysis, a woman’s menopausal status at baseline was determined after reviewing her age at last menstrual period, reason for cessation of menstrual period, hysterectomy status, oophorectomy status, and current MHT use. Women reporting ongoing menstrual periods who had never used hormones for menopausal symptoms were considered premenopausal (*n* = 46,350). Women were classified as perimenopausal (*n* = 2344) if periods had stopped within the last 6 months and they were not currently pregnant, and as postmenopausal (*n* = 55,764) if they met any of the following criteria: (1) periods had stopped for more than 6 months, (2) bilateral oophorectomy, (3) 56 years of age or older at baseline and not already classified as premenopausal or perimenopausal, (4) started using hormone therapy for menopausal symptoms before periods stopped. Women with unknown menopausal status (*n* = 4449) included those who were under age 56 years at baseline and reported having had a hysterectomy on the baseline questionnaire. The age criterion of 56 years as the cutoff for menopause was based on previous work indicating that among women with natural menopause, 97 % were postmenopausal by age 56 years [12]. Among 41,822 peri- or postmenopausal women who had ever used estrogen-only therapy (E only, *n* = 15,729), estrogen plus progesterone combined therapy only (E + P only, *n* = 19,067), or mixed formulations (*n* = 7026), 6723 were baseline-former users, 34,207 were baseline-recent users, and 892 had unknown time of MHT use.

Additional information on demographics, family history of breast cancer, history of a breast biopsy, mammography screening history, age at menarche, detailed history of all pregnancies (including ages at and outcomes for each pregnancy) occurring before baseline, smoking, alcohol consumption at various times in life, height, and weight at age 18 and at baseline, and diet during the year prior to completing the baseline questionnaire (as measured by Block’s food frequency questionnaire) was collected at baseline [13].

### Statistical analyses

Cox proportional hazards models provided multivariable-adjusted estimates of hazard ratios (HRs) and 95 % confidence intervals (CIs) for breast cancer overall and for ER/PR/HER2-defined breast cancer subtypes that were associated with long-term and baseline total, strenuous intensity, and moderate intensity recreational physical activity [14]. In the Cox regression models, the time scale (in days) was defined from age at cohort entry to age at event, censoring, or end of follow-up. Censoring occurred when a participant moved out of California for more than 4 months or died. In addition, in the analysis of invasive breast cancer overall, women with in situ breast cancer were censored on the date of their in situ diagnosis. In the analyses of a specific ER/PR/HER2-defined subtype of invasive breast cancer, women who developed all other subtypes, had an unknown subtype, or had in situ breast cancer were censored on the date of their diagnosis.

We used the same covariates as were used in our previous article [3], except now all models assessing effects of strenuous or moderate recreational physical activity included both strenuous activity and moderate activity (i.e., each was mutually adjusted for the other). The Spearman correlation coefficient was 0.44 for long-term strenuous versus long-term moderate recreational physical activity and 0.34 for baseline strenuous versus baseline moderate activity. Models were stratified by age at baseline (in years) to adjust for calendar effects and were adjusted for race (white, black, Asian, Hispanic, or other/unspecified), family history of breast cancer in a first-degree relative (yes, no, or unknown/adopted), history of a breast biopsy (yes or no), screening mammogram in the 2 years prior to baseline (yes, no, or unknown), BMI at baseline (<25.00, 25.00–29.99, ≥30.00 kg/m^2^, or unknown), combined age at first full-term pregnancy and parity variable (age 15–24 years/1–2 term pregnancies, age 15–24 years/≥3 term pregnancies, age ≥25 years/1–2 term pregnancies, age ≥25 years/≥3 term pregnancies, or nulliparous), combined menopausal status/MHT use (premenopausal; peri-/postmenopausal: no MHT, E only, E + P only, mixed formulations, unknown MHT; or unknown menopausal status), history of smoking at least 100 cigarettes (never, former, current), and alcohol intake during the year prior to baseline (no, yes: <15 g/d, ≥15 g/d, or unknown). Total energy, fat, fiber, antioxidant vitamins, and phytoestrogen intake at baseline [15] and breastfeeding [16] were not included in the multivariable models because they were unrelated to invasive breast cancer risk in this study population.

Tests for trend were conducted to examine the dose-response relationships between recreational physical activity and risk of invasive breast cancer overall and risk of each ER/PR/HER2-defined subtype of invasive breast cancer by fitting the median values of each exposure category to obtain the slope parameter and then determining whether the slope parameter differed from zero using the Wald test [17]. Wald chi-square tests were used to test for homogeneity of trends between TNBC and luminal A-like subtype [18].

For strenuous recreational physical activity, 1-degree of freedom likelihood ratio tests for the homogeneity of two trends were applied to test potential effect modification by BMI at baseline (<25 versus ≥25 kg/m^2^), MHT use at baseline (never versus ever), type of MHT (E only versus E + P), duration of MHT use (<5 versus ≥5 years), or MHT cessation (baseline-former MHT use versus baseline-recent MHT use). Since the effect of BMI differs between premenopausal breast cancer and postmenopausal breast cancer [19, 20], we assessed the potential effect modification of BMI at baseline for strenuous recreational physical activity among all women and among peri- and postmenopausal women at baseline, separately. To avoid small numbers caused by further stratification according to potential effect modifiers, we collapsed the five categories for strenuous recreational physical activity variables into three categories: 0–0.50, 0.51–3.50, and ≥3.51 h/wk/y and used a less detailed variable for race (white versus nonwhite) when analyses were restricted to peri- or postmenopausal women. Additionally, when testing potential effect modification by type, duration, or cessation of MHT use among those who had ever used MHT, these variables were mutually adjusted.

Moreover, in order to assess the impact of changes in strenuous activity between baseline and the 10-year follow-up on the risk of breast cancer overall, we used women who stayed at the lowest level of strenuous recreational physical activity (≤0.50) at both baseline and the 10-year follow-up as reference group and assessed the HRs and 95 % CIs for all other combinations of activity categories (low, ≤0.50 h/wk/y; intermediate, 0.51–3.50 h/wk/y; high, ≥3.51 h/wk/y) across the two time points.

The proportional hazards assumption was examined by fitting a model with an interaction term between each continuous recreational physical activity variable and follow-up time. There was no violation of the assumptions in this study. All *P* values reported are two-sided. We did not adjust *P* values for multiple comparisons. In reporting the results of statistical tests for trend, homogeneity of trends between TNBC and luminal A-like subtype, or effect modification, we considered a two-sided *P* value less than 0.05 as statistically significant. All statistical analyses were performed using SAS version 9.3 software (SAS Institute, Cary, NC, USA).

## Results

### Characteristics

A total of 108,907 women in the analytic cohort were followed for an average of 14.7 years. The age-adjusted distributions of several participant characteristics according to average annual amount of total long-term recreational physical activity are shown in Table 1. Compared with women who were inactive or reported lower levels of total long-term recreational physical activity, those who reported higher levels of total long-term recreational physical activity were more likely to be white, nulliparous, premenopausal, have a lower BMI, and drink alcohol during the year prior to baseline.

**Table 1:** Age-adjusted^a^ percent distribution of baseline characteristics

	Overall, % (*n* = 108,907)	Average annual total long-term RPA (h/wk/y), %				
		≤0.50(*n* = 9984)	0.51–2.50(*n* = 31,942)	2.51–4.50(*n* = 25,442)	4.51–7.00(*n* = 19,466)	≥7.01(*n* = 22,073)
Race						
White	86.4	80.8	85.8	88.4	87.9	87.2
Black	2.7	4.2	3.1	2.4	2.4	2.4
Hispanic	4.5	5.0	4.4	3.9	4.1	4.1
Asian	3.7	7.2	4.4	3.1	2.9	2.8
Others/unspecified	2.8	2.8	2.3	2.2	2.6	3.5
First-degree family history of breast cancer	11.7	12.7	12.5	12.0	11.5	11.3
History of a breast biopsy	15.5	17.0	17.0	15.9	15.4	14.5
Had a mammogram in the 2 years prior to baseline	74.3	84.9	83.1	80.5	77.0	73.1
BMI, kg/m^2^						
<25.00 (under/normal weight)	58.9	51.5	55.4	59.4	61.7	64.0
25.00–29.99 (overweight)	23.9	25.8	24.9	24.3	23.0	21.7
≥30.00 (obese)	13.9	13.9	16.7	13.7	12.9	11.5
Unknown	3.3	4.8	2.9	2.6	2.4	2.8
Nulliparous	26.7	21.7	22.2	23.6	26.5	31.3
Menopausal status and MHT use						
Premenopausal	42.6	26.4	38.5	45.6	49.1	49.7
Peri-/postmenopausal						
No MHT	10.6	15.1	9.7	8.3	7.9	9.1
E only	14.4	17.6	14.0	11.4	10.8	11.3
E plus P only	17.5	22.4	21.5	19.7	17.9	16.1
Mixed formulations	6.5	7.9	6.4	5.6	5.3	5.0
Unknown MHT use	4.3	6.0	4.4	3.8	3.6	3.4
Unknown MP status	4.1	4.6	5.7	5.6	5.4	5.6
History of cigarette smoking						
Never	66.5	66.5	65.8	64.8	65.7	65.7
Former	28.4	27.7	29.0	30.4	29.3	28.5
Current	5.2	5.9	5.2	4.9	5.0	5.8
Alcohol intake during the year prior to baseline						
Nondrinker	31.7	40.4	33.7	29.6	28.7	28.2
<15 g/d	47.9	41.3	47.9	50.6	50.6	50.1
≥15 g/d	15.9	13.4	14.7	16.4	17.1	18.0
Unknown	4.5	4.9	3.7	3.4	3.6	3.7

*Abbreviations*: *BMI* body mass index, *E* estrogen, *g/d* grams/day, *h/wk/y* hour/week/year, *MHT* menopausal hormone therapy, *P* progestin, *RPA* recreational physical activity

Results are for 108,907 women aged less than 80 years in the California Teachers Study followed between 1995 and 2012

^a^Adjusted according to the age distribution (5-year age group) of the 108,907 women who comprise the eligible cohort

### Recreational physical activity and invasive breast cancer

Long-term (from high school till age 54 or age at cohort entry, whichever was younger) and baseline (during the 3 years prior to baseline) total recreational physical activity was inversely associated with breast cancer overall (*P*_trend_ = 0.01 for long-term, *P*_trend_ <0.001 for baseline, Table 2). The analysis modeling strenuous activity and moderate activity as separate variables demonstrated that risk of invasive breast cancer overall was inversely associated with long-term and baseline strenuous recreational physical activity (*P*_trend_ ≤0.03), but no statistically significant association was observed with long-term or baseline moderate recreational physical activity (all *P*_trend_ ≥0.19).

**Table 2:** Adjusted hazard ratios for the associations between recreational physical activity and invasive breast cancer overall and by subtype

h/wk/y	Observed person-years	Overall		TNBC (ER-/PR-/HER2-)^a^		Luminal A-like (ER+ or PR+ plus HER2-)^a^	
		Cases, no.	Adjusted HR (95 % CI)	Cases, no.	Adjusted HR (95 % CI)	Cases, no.	Adjusted HR (95 % CI)
Total (strenuous plus moderate) RPA							
Long-term^c^							
≤0.50	140,148	662	Referent	43	Referent	303	Referent
0.51–2.50	467,293	1923	0.98 (0.89–1.07)	115	0.83 (0.58–1.18)	886	0.95 (0.83–1.08)
2.51–4.50	376,903	1319	0.91 (0.82–1.00)	84	0.80 (0.55–1.16)	589	0.84 (0.73–0.97)
4.51–7.00	289,855	957	0.91 (0.82–1.00)	45	0.58 (0.38–0.89)	445	0.87 (0.75–1.01)
≥7.01	327,027	1021	0.90 (0.81–0.99)	61	0.73 (0.49–1.09)	478	0.87 (0.75–1.00)
*P*_*trend*_			*0.01*		*0.11*		*0.05*
Homogeneity of trends between two subtypes					*P = 0.38*		
Baseline^d^							
≤0.50	355,905	1438	Referent	98	Referent	639	Referent
0.51–2.50	457,799	1708	0.97 (0.90–1.04)	96	0.77 (0.58–1.02)	787	0.99 (0.89–1.10)
2.51–4.50	279,320	972	0.90 (0.83–0.98)	61	0.80 (0.58–1.11)	468	0.96 (0.85–1.08)
4.51–7.00	256,204	901	0.88 (0.81–0.96)	44	0.61 (0.43–0.88)	412	0.89 (0.78–1.01)
≥7.01	251,999	863	0.87 (0.80–0.95)	49	0.72 (0.50–1.02)	395	0.88 (0.77–1.00)
*P*_*trend*_			*<0.001*		*0.07*		*0.01*
Homogeneity of trends between two subtypes					*P = 0.34*		
Strenuous RPA^b^							
Long-term^c^							
≤0.50	449,871	2069	Referent	122	Referent	936	Referent
0.51–2.00	522,886	1 900	0.93 (0.87–0.99)	115	0.89 (0.68–1.16)	865	0.92 (0.84–1.02)
2.01–3.50	293,055	908	0.88 (0.81–0.96)	62	0.93 (0.67–1.29)	430	0.91 (0.80–1.02)
3.51–5.00	160,273	523	0.99 (0.89–1.09)	27	0.76 (0.49–1.19)	237	0.96 (0.83–1.12)
≥5.01	175,143	482	0.87 (0.78–0.97)	22	0.52 (0.31–0.87)	233	0.92 (0.78–1.08)
*P*_*trend*_			*0.03*		*0.02*		*0.35*
Homogeneity of trends between two subtypes					*P = 0.05*		
Baseline^d^							
≤50	894,810	3654	Referent	224	Referent	1675	Referent
0.51–2.00	322,058	1066	0.95 (0.89–1.02)	70	1.00 (0.76–1.32)	478	0.91 (0.82–1.01)
2.01–3.50	157,265	460	0.83 (0.75–0.92)	26	0.73 (0.48–1.11)	216	0.81 (0.70–0.94)
3.51–5.00	153,597	476	0.94 (0.85–1.04)	14	0.42 (0.24–0.73)	229	0.95 (0.83–1.10)
≥5.01	73,497	226	0.90 (0.78–1.04)	14	0.83 (0.47–1.47)	103	0.88 (0.72–1.09)
*P*_*trend*_			*0.01*		*0.01*		*0.06*
Homogeneity of trends between two subtypes					*P = 0.08*		
Moderate RPA^b^							
Long-term^c^							
≤0.50	321,926	1314	Referent	79	Referent	630	Referent
0.51–2.00	582,646	2134	0.99 (0.93–1.07)	134	0.99 (0.74–1.32)	954	0.90 (0.81–1.00)
2.01–3.50	330,827	1175	0.99 (0.91–1.07)	62	0.85 (0.60–1.20)	550	0.93 (0.83–1.05)
3.51–5.00	185,859	637	0.97 (0.88–1.07)	30	0.79 (0.51–1.22)	293	0.89 (0.77–1.03)
≥5.01	179,970	622	0.99 (0.89–1.10)	43	1.34 (0.89–2.02)	274	0.87 (0.74–1.01)
*P*_*trend*_			*0.66*		*0.42*		*0.13*
Homogeneity of trends between two subtypes					*P = 0.20*		
Baseline^d^							
≤0.50	519,391	1937	Referent	128	Referent	884	Referent
0.51–2.00	503,498	1788	1.00 (0.93–1.07)	99	0.82 (0.62–1.07)	808	0.98 (0.89–1.09)
2.01–3.50	233,772	891	1.04 (0.96–1.13)	51	0.93 (0.66–1.30)	438	1.11 (0.99–1.25)
3.51–5.00	226,867	799	0.91 (0.83–0.99)	43	0.83 (0.58–1.19)	366	0.90 (0.80–1.03)
≥5.01	117,700	467	0.98 (0.88–1.09)	27	0.98 (0.63–1.54)	205	0.95 (0.80–1.11)
*P*_*trend*_			*0.19*		*0.75*		*0.32*
Homogeneity of trends between two subtypes					*P = 0.98*		

HRs are from multivariable Cox proportional hazards regression models using age (in days) as the time metric and stratified by age (in years) with the adjustment for race, family history of breast cancer in first-degree relatives, combined age at first full-term pregnancy and parity variable, combined menopausal status and MHT use variable, BMI at baseline, history of smoking, alcohol intake during the past year of baseline, screening mammogram in the past 2 years of baseline, and history of a breast biopsy

*Abbreviations*: *BMI* body mass index, *CI* confidence interval, *ER* estrogen receptor, *HER2* human epidermal growth factor receptor 2, *HR* hazard ratio, *h/wk/y* hour/week/year, *MHT* menopausal hormone therapy, *PR* progesterone receptor, *RPA* recreational physical activity, *TNBC* triple negative breast cancer

^a^Diagnosed in 1999 and afterward

^b^Additionally, strenuous RPA and moderate RPA mutually adjusted

^c^RPA from high school till age 54 or age at cohort entry, whichever was younger

^d^RPA during the 3 years prior to baseline

Analyses for ER/PR/HER2-defined subtypes showed that both long-term and baseline total recreational physical activity were associated with lower risk of TNBC and luminal A-like subtype, although the trend tests for TNBC did not reach statistical significance (*P*_trend_ <0.11 for TNBC, *P*_trend_ ≤0.05 for luminal A). The analysis modeling strenuous activity and moderate activity as separate variables showed that the inverse associations with long-term and baseline strenuous recreational physical activity were confined to women with TNBC (*P*_trend_ ≤0.02); the trends in risk of TNBC were marginally different from those for luminal A-like subtype for both long-term activity (*P*_homogeneity of trends_ = 0.05) and baseline activity (*P*_homogeneity of trends_ = 0.08). The RRs (95 % CIs) comparing the highest (≥5.01 h/wk/y) to the lowest category (≤0.50 h/wk/y) of long-term strenuous physical activity were 0.52 (0.31–0.87) for TNBC and 0.92 (0.78–1.08) for luminal A-like subtype.

No statistically significant association was observed for long-term or baseline moderate recreational physical activity with either TNBC or luminal-A subtype (all *P*_trend_ ≥0.13). Moreover, we did not observe any evidence that recreational physical activity was associated with luminal B-like or HER2-enriched breast cancer (Additional file 1).

### Potential interaction between strenuous recreational physical activity and BMI at baseline

Among all women, we did not observe any evidence that BMI modified the association between strenuous recreational physical activity and TNBC or luminal A-like subtype (*P*_homogeneity of trends_ ≥0.31, Table 3). Risk of TNBC was inversely associated with baseline strenuous recreational physical activity among heavier peri- or postmenopausal women (BMI ≥25 kg/m^2^, *P*_trend_ = 0.02) but not among their leaner counterparts (BMI <25 kg/m^2,^*P*_trend_ = 0.42); however, the test for homogeneity of trends did not reach statistical significance (*P*_homogeneity of trends_ = 0.06).

**Table 3:** Adjusted hazard ratios for the associations between strenuous recreational physical activity and invasive breast cancer subtypes by body mass index at baseline

BMI, kg/m^2^	Cases, no.	Strenuous RPA, h/wk/y			*P*_*trend*_	*P*_homogeneity of trends_
		≤0.50	0.51–3.50	≥3.51		
		Adjusted HR (95 % CI)	Adjusted HR (95 % CI)	Adjusted HR (95 % CI)		
Among all women						
TNBC (ER-/PR-/HER2-)						
Long-term^a^						
BMI <25	195	Referent	0.88 (0.64–1.22)	0.62 (0.39–0.99)	*0.05*	*0.54*
BMI ≥25	139	Referent	0.85 (0.59–1.24)	0.78 (0.45–1.36)	*0.37*	
Baseline^b^						
BMI <25	195	Referent	1.04 (0.76–1.42)	0.60 (0.37–0.97)	*0.05*	*0.40*
BMI ≥25	139	Referent	0.72 (0.47–1.10)	0.48 (0.21–1.10)	*0.04*	
Luminal A-like (ER+ or PR+ plus HER2-)						
Long-term^a^						
BMI <25	1484	Referent	0.95 (0.84–1.07)	0.93 (0.79–1.09)	*0.39*	*0.71*
BMI ≥25	1119	Referent	0.87 (0.76–0.99)	0.90 (0.74–1.08)	*0.25*	
Baseline^b^						
BMI <25	1484	Referent	0.89 (0.79–1.00)	0.88 (0.76–1.03)	*0.08*	*0.31*
BMI ≥25	1119	Referent	0.87 (0.75–1.00)	1.06 (0.86–1.32)	*0.99*	
Among peri- and postmenopausal women at baseline						
TNBC (ER-/PR-/HER2-)						
Long-term^a^						
BMI <25	118	Referent	1.01 (0.67–1.53)	0.78 (0.41–1.47)	*0.45*	*0.99*
BMI ≥25	89	Referent	0.89 (0.56–1.43)	0.83 (0.40–1.73)	*0.60*	
Baseline^b^						
BMI <25	118	Referent	1.26 (0.83–1.90)	0.69 (0.35–1.36)	*0.42*	*0.06*
BMI ≥25	89	Referent	0.56 (0.31–1.02)	0.28 (0.07–1.17)	*0.02*	
Luminal A-like (ER+ or PR+ plus HER2-)						
Long-term^a^						
BMI <25	940	Referent	0.97 (0.84–1.12)	0.90(0.73–1.12)	*0.34*	*0.39*
BMI ≥25	794	Referent	0.86 (0.73–1.00)	0.80(0.63–1.02)	*0.06*	
Baseline^b^						
BMI <25	940	Referent	0.93 (0.80–1.09)	0.86 (0.70–1.05)	*0.13*	*0.47*
BMI ≥25	794	Referent	0.90 (0.75–1.07)	1.01 (0.77–1.32)	*0.78*	

HRs are from multivariable Cox proportional hazards regression models using age (in days) as the time metric and stratified by age (in years) with the adjustment for race, family history of breast cancer in first-degree relatives, combined age at first full-term pregnancy and parity variable, combined menopausal status and MHT use variable, history of smoking, alcohol intake during the past year of baseline, screening mammogram in the past 2 years of baseline, history of a breast biopsy, and a corresponding moderate RPA variable

*Abbreviations*: *BMI* body mass index, *CI* confidence interval, *ER* estrogen receptor, *HER2* human epidermal growth factor receptor 2, *HR* hazard ratio, *h/wk/y* hour/week/year, *MHT* menopausal hormone therapy, *PR* progesterone receptor, *RPA* recreational physical activity, *TNBC* triple negative breast cancer

^a^RPA from high school till age 54 or age at cohort entry, whichever was younger

^b^RPA during the 3 years prior to baseline

### Potential interaction between strenuous recreational physical activity and MHT use variables among women who were peri- or postmenopausal at baseline

MHT use (never versus ever) did not modify the associations between long-term or baseline strenuous physical activity and risk of TNBC or luminal A-like subtype (all *P*_homogeneity of trends_ ≥0.15, Table 4). Moreover, MHT cessation (baseline-former MHT use versus baseline-recent MHT use) did not modify the observed associations of TNBC with long-term or baseline strenuous recreational physical activity (*P*_homogeneity of trends for baseline-former versus baseline-recent MHT use_ ≥0.30, Table 5). However, MHT cessation modified the effects of strenuous recreational physical activity on luminal A-like subtype (*P*_homogeneity of trends for baseline-former versus baseline-recent MHT use_: 0.03 and 0.02 for long-term and baseline strenuous recreational physical activity, respectively). The inverse associations with both long-term and baseline strenuous recreational physical activity were observed among baseline-former MHT users (both *P*_trend_ = 0.02), but not among women who were baseline-recent MHT users (*P*_trend_ ≥0.59). Former MHT users in the highest category (≥3.51 h/wk/y) of long-term strenuous recreational physical activity had 48 % lower risk of luminal A-like subtype when compared to their counterparts in the lowest category (≤0.50 h/wk/y) (HR = 0.52, 95 % CI = 0.30–0.89); similarly, former MHT users in the highest versus lowest category of baseline strenuous recreational physical activity had 56 % lower risk of luminal A-like breast cancer subtype (HR = 0.44, 95 % CI = 0.22–0.87).

**Table 4:** Adjusted hazard ratios for the associations between strenuous recreational physical activity and invasive breast cancer subtypes by menopausal hormone therapy use

	Cases, no.	Strenuous RPA, h/wk/y			*P*_*trend*_	*P*_homogeneity of trends_
		≤0.50	0.51–3.50	≥3.51		
		Adjusted HR (95 % CI)	Adjusted HR (95 % CI)	Adjusted HR (95 % CI)		
TNBC (ER-/PR-/HER2-)						
Long-term^a^						
Never MHT use at baseline	37	Referent	1.43 (0.72–2.84)	0.35 (0.08–1.56)	*0.23*	*0.32*
Ever MHT use at baseline	165	Referent	0.93 (0.66–1.32)	0.91 (0.54–1.52)	*0.69*	
Baseline^b^						
Never MHT use at baseline	37	Referent	0.92 (0.43–1.98)	0.43 (0.10–1.83)	*0.26*	*0.65*
Ever MHT use at baseline	165	Referent	1.00 (0.70–1.44)	0.60 (0.32–1.13)	*0.14*	
Luminal A-like (ER+ or PR+ plus HER2-)						
Long-term^a^						
Never MHT use at baseline	315	Referent	0.92 (0.72–1.17)	0.86 (0.61–1.20)	*0.36*	*0.75*
Ever MHT use at baseline	1357	Referent	0.92 (0.81–1.04)	0.91 (0.76–1.09)	*0.28*	
Baseline^b^						
Never MHT use at baseline	315	Referent	0.83 (0.63–1.09)	0.73 (0.50–1.07)	*0.07*	*0.15*
Ever MHT use at baseline	1357	Referent	0.92 (0.81–1.05)	0.97 (0.81–1.16)	*0.57*	

Results are for 53,394 peri- and postmenopausal women who never used MHT or ever used E only, E + P only or mixed formulations at baseline. HRs are from multivariable Cox proportional hazards regression models using age (in days) as the time metric and stratified by age (in years) with the adjustment for race, family history of breast cancer in first-degree relatives, combined age at first full-term pregnancy and parity variable, BMI at baseline, history of smoking, alcohol intake during the past year of baseline, screening mammogram in the past 2 years of baseline, history of a breast biopsy, and a corresponding moderate RPA variable

*Abbreviations*: *BMI* body mass index, *CI* confidence interval, *E* estrogen, *ER* estrogen receptor, *HER2* human epidermal growth factor receptor 2, *HR* hazard ratio, *h/wk/y* hour/week/year, *MHT* menopausal hormone therapy, *P* progestin, *PR* progesterone receptor, *RPA* recreational physical activity, *TNBC* triple negative breast cancer

^a^RPA from high school till age 54 or age at cohort entry, whichever was younger

^b^RPA during the 3 years prior to baseline

**Table 5:** Adjusted hazard ratios for the associations between strenuous recreational physical activity and invasive breast cancer subtypes among menopausal hormone therapy users

	Cases, no.	Strenuous RPA, h/wk/y			*P*_*trend*_	*P*_homogeneity of trends_
		≤0.50	0.51–3.50	≥3.51		
		Adjusted HR (95 % CI)	Adjusted HR (95 % CI)	Adjusted HR (95 % CI)		
TNBC (ER-/PR-/HER2-)						
Long-term^a^						
Type of MHT use						
E only	47	Referent	0.80 (0.43–1.48)	0.63 (0.23–1.71)	*0.34*	*0.29*
E + P	108	Referent	0.99 (0.64–1.52)	1.12 (0.61–2.08)	*0.71*	
Duration of MHT use						
<5 years	80	Referent	0.98 (0.60–1.62)	1.09 (0.55–2.19)	*0.80*	*0.53*
≥5 years	75	Referent	0.87 (0.53–1.42)	0.81 (0.37–1.75)	*0.57*	
Cessation of MHT use						
Former MHT use	24	Referent	1.00 (0.42–2.38)	0.81 (0.22–3.02)	*0.77*	*0.81*
Recent MHT use	131	Referent	0.91 (0.61–1.34)	0.98 (0.55–1.74)	*0.92*	
Baseline^b^						
Type of MHT use						
E only	47	Referent	0.93 (0.47–1.87)	0.89 (0.31–2.56)	*0.80*	*0.60*
E + P	108	Referent	0.98 (0.63–1.52)	0.58 (0.26–1.28)	*0.20*	
Duration of MHT use						
<5 years	80	Referent	1.00 (0.61–1.66)	0.46 (0.16–1.28)	*0.17*	*0.40*
≥5 years	75	Referent	0.93 (0.54–1.60)	0.90 (0.40–2.02)	*0.76*	
Cessation of MHT use						
Former MHT use	24	Referent	0.58 (0.20–1.72)	0.35 (0.05–2.64)	*0.21*	*0.30*
Recent MHT use	131	Referent	1.05 (0.70–1.56)	0.73 (0.37–1.44)	*0.43*	
Luminal A-like (ER+ or PR+ plus HER2-)						
Long-term^a^						
Type of MHT use						
E only	375	Referent	1.00 (0.80–1.25)	0.84 (0.60–1.17)	*0.31*	*0.75*
E + P	931	Referent	0.89 (0.77–1.02)	0.90 (0.73–1.11)	*0.32*	
Duration of MHT use						
<5 years	580	Referent	0.81 (0.67–0.97)	0.90 (0.70–1.16)	*0.41*	*0.89*
≥5 years	726	Referent	1.01 (0.86–1.19)	0.86 (0.67–1.10)	*0.26*	
Cessation of MHT use						
Former MHT use	171	Referent	0.84 (0.61–1.16)	0.52 (0.30–0.89)	*0.02*	*0.03*
Recent MHT use	1135	Referent	0.93 (0.82–1.06)	0.95 (0.78–1.15)	*0.59*	
Baseline^b^						
Type of MHT use						
E only	375	Referent	0.82 (0.63–1.06)	0.94 (0.67–1.32)	0.49	0.65
E + P	931	Referent	0.96 (0.82–1.12)	0.98 (0.79–1.21)	0.75	
Duration of MHT use						
<5 years	580	Referent	0.91 (0.75–1.11)	0.86 (0.65–1.14)	*0.24*	*0.29*
≥5 years	726	Referent	0.92 (0.77–1.10)	1.05 (0.83–1.33)	*0.83*	
Cessation of MHT use						
Former MHT use	171	Referent	0.96 (0.67–1.36)	0.44 (0.22–0.87)	*0.02*	*0.02*
Recent MHT use	1135	Referent	0.92 (0.79–1.06)	1.05 (0.87–1.27)	*0.84*	

Results are for 39,715 peri- and postmenopausal women who had ever used MHT with known information regarding type, duration and cessation of MHT use at baseline. HRs are from multivariable Cox proportional hazards regression models using age (in days) as the time metric and stratified by age (in years) with the adjustment for race, family history of breast cancer in first-degree relatives, combined age at first full-term pregnancy and parity variable, BMI at baseline, history of smoking, alcohol intake during the past year of baseline, screening mammogram in the past 2 years of baseline, history of a breast biopsy, and a corresponding moderate RPA variable; additionally, type of MHT, duration of MHT, and cessation of MHT were mutually adjusted

*Abbreviations*: *BMI* body mass index, *CI* confidence interval, *E* estrogen, *ER* estrogen receptor, *HER2* human epidermal growth factor receptor 2, *HR* hazard ratio, *h/wk/y* hour/week/year, *MHT* menopausal hormone therapy, *P* progestin, *PR* progesterone receptor, *RPA* recreational physical activity, *TNBC* triple negative breast cancer

^a^RPA from high school till age 54 or age at cohort entry, whichever was younger

^b^RPA during the 3 years prior to baseline

### Changes in the levels of strenuous recreational physical activity and breast cancer risk

Compared to women who stayed at the lowest level of strenuous recreational physical activity (≤0.50 h/wk/y) between baseline and the 10-year follow-up, the lowest HR was observed among those who consistently engaged in the highest level of strenuous recreational physical activity (≥3.51 h/wk/y) in adulthood (HR = 0.71, 95 % CI = 0.52–0.98, Table 6). A lower HR was also observed among women who decreased strenuous recreational physical activity from the highest level at baseline to an intermediate level (0.51–3.50 h/wk/y) at 10-year follow-up (HR = 0.75, 95 % CI = 0.55–1.02), but this was not observed among those who decreased to the lowest level at 10-year follow-up (HR = 1.00, 95 % CI = 0.76–1.32).

**Table 6:** Adjusted hazard ratios for the associations between changes in strenuous recreational physical activity levels and invasive breast cancer

RPA at baseline^a^, h/wk/y	RPA at 10-year follow-up^b^, h/wk/y	Overall	Observed person-years	Cases, no.	Adjusted HR (95 % CI)
Breast cancer overall					
No change					
Low (≤0.50)	Low (≤0.50)	24,028	396,185	692	Referent
Intermediate (0.51–3.50)	Intermediate (0.51–3.50)	6273	104,479	155	1.01 (0.84–1.21)
High (≥3.51)	High (≥3.51)	2462	41,105	42	0.71 (0.52–0.98)
Increasing					
Low (≤0.50)	Intermediate (0.51–3.50)	5640	93,840	127	0.86 (0.71–1.04)
Low (≤0.50)	High (≥3.51)	1267	21,076	34	1.03 (0.72–1.45)
Intermediate (0.51–3.50)	High (≥3.51)	1751	29,121	40	0.89 (0.64–1.23)
Decreasing					
Intermediate (0.51–3.50)	Low (≤0.50)	8436	139,718	209	0.96 (0.82–1.12)
High (≥3.51)	Low (≤0.50)	2212	36,515	58	1.00 (0.76–1.32)
High (≥3.51)	Intermediate (0.51–3.50)	2617	43,519	46	0.75 (0.55–1.02)

Results are for 54,686 women who completed baseline and 10-year follow-up questionnaires regarding RPA in recent 3 years in the California Teachers Study, 1995–2012. HRs are from multivariable Cox proportional hazards regression models using age (in days) as the time metric and stratified by age (in years) with the adjustment for race, family history of breast cancer in first-degree relatives, combined age at first full-term pregnancy and parity variable, combined menopausal status and MHT use variable, BMI at baseline, history of smoking, alcohol intake during the past year of baseline, screening mammogram in the past 2 years of baseline, history of a breast biopsy, and a corresponding changes in level of moderate RPA variable

*Abbreviations*: *BMI* body mass index, *CI* confidence interval, *HR* hazard ratio, *h/wk/y* hour/week/year, *MHT* menopausal hormone therapy, *RPA* recreational physical activity

^a^RPA during the 3 years prior to baseline

^b^RPA during the 3 years prior to 10-year follow-up

## Discussion

In this cohort of 108,907 women who were followed for an average of 14.7 years, both long-term and baseline total recreational physical activity and strenuous intensity physical activity, but not moderate intensity physical activity, were inversely associated with risk of invasive breast cancer. The results were similar to those we previously reported based on 7-year follow-up data [3], except that the statistically significant inverse associations with baseline total and strenuous recreational physical activity were observed only in the current analyses. Analyses of ER/PR/HER2-defined subtypes showed that both long-term and baseline total recreational physical activity were associated with lower risk of TNBC and luminal A-like subtype, but recreational physical activity was not associated with the other two subtypes. Furthermore, when strenuous activity and moderate activity were modeled as separate variables, the inverse association with long-term and baseline strenuous physical activity was confined to TNBC.

TNBC is the second most common breast cancer subtype following luminal A-like breast cancer, accounting for 10 to 25 % of invasive cases [21, 22]. TNBC is characterized by its biological aggressiveness and poor prognosis compared to luminal A-like subtype [22–24]. Unlike ER+ or HER2+ breast cancers, which, respectively, usually respond to anti-estrogen or trastuzumab treatment, TNBC has no effective targeted therapies. It is important to determine whether recreational physical activity lowers risk of TNBC, as such findings would provide epidemiologic evidence for developing a preventive approach for TNBC.

TNBC is characterized by a basal-like molecular profile, exhibiting overexpression of genes involved in cell proliferation and differentiation, and p21-mediated G1-S checkpoints of cell cycle signaling pathways. Luminal A-like breast cancer is characterized by luminal molecular profiles associated with the ER signaling pathway [25, 26]. The inverse association of recreational physical activity with breast cancer may differ between these two subtypes if recreational physical activity influences these signaling pathways differently.

Most previous epidemiologic studies have been limited in ability to assess associations between recreational physical activity and breast cancer subtypes defined by ER status or ER and PR status jointly [3, 27–50]. The majority of these studies found that recreational physical activity was associated with a lower risk of breast cancer regardless of ER or ER/PR status [27–42, 49]. Five studies reported statistically significant association only among women with ER+ or ER+/PR+ breast cancer [43–47], whereas three studies found risk reduction limited to women with ER– or ER–/PR– breast cancer [3, 48, 50]. Only three studies to date have investigated the association between recreational physical activity and TNBC [4–6]. The Women’s Health Initiative Cohort Study reported that risks of both TNBC and ER+ breast cancer were inversely associated with recreational physical activity among postmenopausal women [4]. Results from a population-based case-control study of women aged 20–54 years have shown that engaging in levels of recreational physical activity above the median in the year before the interview was associated with a lower risk for both TNBC and the luminal A-like subtype [5]. In the Women’s Contraceptive and Reproductive Experiences (CARE) Study, lifetime recreational physical activity reduced risk of both TNBC and the luminal A-like subtype, but not HER2+ subtypes [6]. The results presented here, along with the published findings of three previous studies [4–6], show that recreational physical activity reduces risk of both TNBC and luminal A-like subtype. Our data additionally showed that the protective effect of strenuous physical activity was confined to TNBC.

Many previous studies have assessed whether the association between recreational physical activity and breast cancer risk is modified by BMI [32, 33, 35, 49–64]. The majority of studies assessing risk of all breast cancers did not show that the risk reduction achieved with recreational physical activity was modified by BMI [35, 49, 51–56, 59]; a small number of studies reported that the association of recreational physical activity and breast cancer risk was more evident among lean postmenopausal women than overweight postmenopausal women [32, 33, 57, 60–62]. Three studies observed a more pronounced inverse association between recreational physical activity and the risk of breast cancer overall for overweight or obese postmenopausal women than for their leaner counterparts [50, 63, 64]; further, in one study, BMI effect modification was restricted to ER– or ER–/PR– subtype of breast cancer [50]. At this time, it appears that this analysis is the first to investigate whether the reduction in risk of TNBC associated with strenuous recreational physical activity is modified by BMI. It showed that no statistically significant effect modification by BMI; however, the risk reduction associated with baseline strenuous recreational physical activity was statistically significant among overweight or obese peri- or postmenopausal women, but not among their leaner counterparts.

Moreover, in the CTS, the association between recreational physical activity and breast cancer risk was similar among users and nonusers of MHT, which is consistent with most previous studies [3, 33, 53, 57, 58, 65–67]. In contrast, in the U.S. Radiologic Technologist cohort, recreational physical activity in the year prior to baseline was inversely associated with breast cancer risk among postmenopausal women who had never used MHT at baseline, but not among those who had ever used MHT [68]. Only a few studies have examined the association for breast cancer overall with recreational physical activity according to a woman’s more detailed history of MHT use [32, 60, 69]. In the American Cancer Society Cancer Prevention Study II Nutrition Cohort, the inverse association between recreational physical activity and risk of breast cancer appeared to be stronger among women who were not using MHT at baseline than among those who used MHT at baseline [60]. One population-based case-control study reported an inverse association between breast cancer risk and lifetime total of vigorous recreational physical activity that was restricted to postmenopausal women who had no recent exposure (within the past 2 years) to MHT [32]. The Women’s CARE Study reported inverse associations between recreational physical activity and breast cancer risk in the following groups: women who had never used MHT, women who formerly used MHT, and women who currently used E only, but no association was observed in women who currently used E + P [69]. Our findings add to this literature, showing that the effect modification by cessation of MHT is restricted to the luminal A-like subtype of breast cancer and that recreational physical activity is inversely associated with risk of TNBC regardless of a woman’s history of MHT use.

In addition, although participation in recreational physical activity may change over time in adulthood, only two prospective studies have investigated whether these changes influence the observed associations with breast cancer [42, 49]. In the Nurses’ Health Study, postmenopausal women who were active (≥9 MET-h/wk) close to baseline but not 20 years later did not have a lower risk of breast cancer compared to postmenopausal women who were less active (<9 MET-h/week) during both periods, whereas those who were active 20 years later had a 10 % lower risk, regardless of whether or not they were active around baseline, suggesting that recent recreational physical activity is more important than activity done many years earlier for reducing breast cancer risk [42]. Analysis using the follow-up data between 1993 and 2005 collected by the French component of the European Prospective Investigation into Cancer and Nutrition (E3N) cohort showed that women with a higher level of recent recreational physical activity (≥12 MET-h/wk, self-reported in 2002) had lower risk of breast cancer than those with a lower level of recent recreational physical activity (<12 MET-h/wk), regardless of their recreational physical activity level at baseline (self-reported in 1993) [49]. Furthermore, they demonstrated that compared to women who remained physically active (≥12 MET-h/wk) from baseline to the 2005 data collection, those with decreased levels of recent activity (<12 MET-h/wk) had an increased risk of breast cancer (HR = 1.16; 95 % CI: 1.01–1.35), which suggests that consistently engaging in high-intensity recreational physical activity is important for reducing breast cancer risk [49]. Consistent with these findings from the E3N cohort, we found that the lowest risk estimate for breast cancer was among women who consistently engaged in higher levels of strenuous recreational physical activity between baseline and the 10-year follow-up.

This study has several limitations. First, the information on ER, PR, and HER2 status was collected by the regional registries in California from pathology laboratories located throughout the state; these laboratories may vary in their application of methods and the cut points used to assign a positive status. Nevertheless, it is unlikely that any methodological differences would influence the observed associations. A large validation study comparing registry reports of ER and PR status to those of a single “expert” laboratory showed high concordance for ER+/PR+ and ER–/PR– and only small differences in risk estimates for ER+/PR+ and ER–/PR– breast cancers [70]. Second, not all women with invasive breast cancer had ER, PR, or HER2 status available. However, the number of breast cancer patients in our study with missing ER or PR status was relatively small (7.8 % missing/unknown ER status; 9.1 % missing/unknown PR status) and lower than the rates presented in previous studies based on SEER registry data [71, 72]. Among women diagnosed with invasive breast cancer since 1999, the percentage of women with missing HER2 status (24.8 %) is also lower than the rates reported in previous studies of SEER data [9, 73]. It is worthy to note that the percentage of women with missing HER2 status was higher when HER2 status was first introduced (51.3 % in 1999 and 43.9 % in 2000), which would mean that cases in those 2 years are underrepresented in our analyses by receptor subtype. We therefore repeated our analyses for the ER/PR/HER2-defined specific subtypes using cases diagnosed from 2001 through 2012 and found the risk estimates were similar to those based on the cases diagnosed from 1999 through 2012. We also compared both continuous and categorical representations of strenuous recreational physical activity between case patients with and without known ER, between case patients with and without known PR, between case patients with and without known HER2 among all case patients and among those diagnosed from 1999 onward. No statistically significant differences were detected for any of these comparisons (all *P*_t test_ ≥0.10, all *P*_χ_^2^ ≥0.22, data not shown). Third, we were unable to determine whether our findings regarding change in recreational physical activity from baseline to the 10-year follow-up differ by tumor receptor status because of the reduced number of breast cancer patients in those analyses. In addition, we had only one additional recreational physical activity measurement after our baseline assessment. It is possible that the 10-year follow-up assessment did not reflect women’s activity during the 10-year interval between the two assessments. It is likely that this misclassification is nondifferential since our analyses of changes in women’s physical activity levels is only based on invasive breast cancers that were diagnosed after women submitted their 10-year follow-up questionnaires. Fourth, we did not collect information on occupational physical activity in the CTS. The CTS cohort consists of women who were active, recently active or retired teachers and administrators at baseline; we would not expect substantial variation in occupational activity among women who were active in the California public school system. However, women may have retired during follow-up or engaged in other occupations; unfortunately we do not have information on other occupations held. Moreover, we did not collect information regarding long-term household physical activity or long-term sedentary behavior, whereas we have baseline data on average hours per day per week (h/d/wk) a woman spent doing housework or sitting within the past year. Inclusion of these factors in our models assessing baseline recreational physical activity did not alter our findings (data not shown). Finally, we did not have activity-specific information on intensity; but we did collect information on two intensity levels of activity, strenuous or moderate physical activity, by providing examples of activities for each intensity level. Although it is possible that a woman may have incorrectly reported her activities as strenuous or moderate thereby overestimating or underestimating the amount of energy she expended, the information on physical activity was collected before breast cancers were diagnosed and reporting of activities as strenuous or moderate should not differ by disease status overall or by receptor status of the tumor.

Despite these potential limitations, this study has several strengths. First, by collecting information on recreational physical activity and potential effect modifiers at baseline (prior to diagnosis), differential recall by disease status, which may occur in case-control studies, was minimized. Second, the CTS utilizes detailed follow-up procedures that result in virtually complete ascertainment of cancer outcomes. Third, a large number of invasive breast cancers were accrued during follow-up of the 108,907 women included in the analyses presented here giving us the ability to assess the effects of both long-term and baseline recreational physical activity on different tumor subtypes. Fourth, updated information on recreational physical activity from the 10-year follow-up questionnaire enabled us to examine whether changes during adulthood in the amount of recreational physical activity influence breast cancer risk. Finally, we believe that our study is the first to examine the possible interactions between recreational physical activity, BMI and type, duration and cessation of MHT use on risk of ER/PR/HER2-defined breast cancer subtypes.

## Conclusions

Our data suggest that strenuous recreational physical activity, either long-term or in recent adulthood, may reduce a woman’s risk of breast cancer, especially the TNBC subtype. Because few known risk or protective factors for breast cancer are modifiable, our findings highlight the public health importance of promoting physically active lifestyles to reduce women’s risk of TNBC.

## Abbreviations

BMI, body mass index; CARE, Contraceptive and Reproductive Experiences; CCR, California Cancer Registry; CI, confidence interval; CTS, California Teachers Study; E, estrogen; E3N, European Prospective Investigation into Cancer and Nutrition; ER, estrogen receptor; h/wk/y, hour/week/year; HER2, human epidermal growth factor receptor 2; HR, hazard ratio; MHT, menopausal hormone therapy; P, progestin; PR, progesterone receptor; SEER, Surveillance, Epidemiology, and End Results; TNBC, triple negative breast cancer

## Additional file

Additional file 1: Table S1. Adjusted hazard ratios for the associations between recreational physical activity and luminal B-like and HER2-enriched invasive breast cancer. (DOCX 22 kb)
