# Supplementary material for: Recreational physical activity and risk of triple negative breast cancer in the California Teachers Study
Source: Breast Cancer Res. 2016 Jun 17;18:62. doi: 10.1186/s13058-016-0723-3 (PMC4912767; doi:10.1186/s13058-016-0723-3)
Supplement: Additional file 1: Table S1. — Adjusted hazard ratios for the associations between recreational physical activity and luminal B-like and HER2-enriched invasive breast cancer. (DOCX 22 kb) [file 13058_2016_723_MOESM1_ESM.docx]

**Table S1**. Adjusted hazard ratios for the associations between recreational physical activity and luminal B-like and HER2-enriched invasive breast cancer

| h/wk/y | | Observed  Person-years | Luminal B-like  (ER+ or PR+ plus HER2+)^a^ | | HER2-enriched  (ER-/PR-/HER2+)^a^ | |
| --- | --- | --- | --- | --- | --- | --- |
|  |  |  | Cases | Adjusted HR | Cases | Adjusted HR |
|  |  |  | No. | (95% CI) | No. | (95% CI) |
| Total (strenuous plus moderate) RPA | | |  |  |  |  |
|  | Long-term^c^ |  |  |  |  |  |
|  | ≤0.50 | 140148 | 33 | Referent | 15 | Referent |
|  | 0.51-2.50 | 467293 | 117 | 1.13(0.77-1.67) | 53 | 1.15(0.64-2.05) |
|  | 2.51-4.50 | 376903 | 103 | 1.30(0.87-1.94) | 32 | 0.91(0.49-1.70) |
|  | 4.51-7.00 | 289855 | 68 | 1.17(0.77-1.79) | 29 | 1.12(0.59-2.12) |
|  | ≥7.01 | 327027 | 72 | 1.16(0.76-1.77) | 30 | 1.08(0.57-2.03) |
|  | *P_trend_* |  |  | *0.75* |  | *0.97* |
|  | Baseline^d^ |  |  |  |  |  |
|  | ≤0.50 | 355905 | 90 | Referent | 46 | Referent |
|  | 0.51-2.50 | 457799 | 129 | 1.16(0.88-1.52) | 40 | 0.69(0.45-1.06) |
|  | 2.51-4.50 | 279320 | 63 | 0.95(0.69-1.32) | 22 | 0.61(0.36-1.02) |
|  | 4.51-7.00 | 256204 | 63 | 1.03(0.74-1.43) | 28 | 0.82(0.51-1.33) |
|  | ≥7.01 | 251999 | 48 | 0.82(0.57-1.18) | 23 | 0.70(0.42-1.18) |
|  | *P_trend_* |  |  | *0.14* |  | *0.41* |
| Strenuous RPA^b^ | | |  |  |  |  |
|  | Long-term^c^ |  |  |  |  |  |
|  | ≤0.50 | 449871 | 123 | Referent | 57 | Referent |
|  | 0.51-2.00 | 522886 | 134 | 1.00(0.77-1.29) | 45 | 0.72(0.48-1.08) |
|  | 2.01-3.50 | 293055 | 63 | 0.88(0.64-1.22) | 23 | 0.74(0.44-1.23) |
|  | 3.51-5.00 | 160273 | 35 | 0.95(0.63-1.41) | 19 | 1.22(0.69-2.14) |
|  | ≥5.01 | 175143 | 38 | 1.01(0.67-1.52) | 15 | 1.04(0.55-1.96) |
|  | *P_trend_* |  |  | *0.85* |  | *0.60* |
|  | ≤0.50 | 894810 | 240 | Referent | 104 | Referent |
|  | Baseline ^d^ |  |  |  |  |  |
|  | 0.51-2.00 | 322058 | 78 | 1.00(0.77-1.30) | 21 | 0.60(0.37-0.97) |
|  | 2.01-3.50 | 157265 | 30 | 0.84(0.57-1.24) | 10 | 0.60(0.31-1.16) |
|  | 3.51-5.00 | 153597 | 29 | 0.85(0.57-1.27) | 15 | 0.98(0.56-1.72) |
|  | ≥5.01 | 73497 | 16 | 1.04(0.61-1.77) | 9 | 1.28(0.61-2.66) |
|  | *P_trend_* |  |  | *0.58* |  | *0.99* |
| Moderate RPA^b^ | | |  |  |  |  |
|  | Long-term^c^ |  |  |  |  |  |
|  | ≤0.50 | 321926 | 81 | Referent | 30 | Referent |
|  | 0.51-2.00 | 582646 | 143 | 1.03(0.77-1.36) | 64 | 1.36(0.87-2.13) |
|  | 2.01-3.50 | 330827 | 82 | 1.08(0.78-1.49) | 33 | 1.24(0.74-2.08) |
|  | 3.51-5.00 | 185859 | 46 | 1.11(0.76-1.62) | 19 | 1.20(0.65-2.20) |
|  | ≥5.01 | 179970 | 41 | 1.01(0.67-1.53) | 13 | 0.81(0.40-1.64) |
|  | *P_trend_* |  |  | *0.83* |  | *0.43* |
|  | Baseline ^d^ |  |  |  |  |  |
|  | ≤0.50 | 519391 | 134 | Referent | 51 | Referent |
|  | 0.51-2.00 | 503498 | 136 | 1.09(0.85-1.40) | 53 | 1.22(0.82-1.81) |
|  | 2.01-3.50 | 233772 | 46 | 0.81(0.57-1.15) | 21 | 1.00(0.59-1.69) |
|  | 3.51-5.00 | 226867 | 52 | 0.94(0.67-1.31) | 23 | 1.03(0.61-1.72) |
|  | ≥5.01 | 117700 | 25 | 0.84(0.53-1.32) | 11 | 0.85(0.42-1.71) |
|  | *P_trend_* |  |  | *0.27* |  | *0.61* |

HRs are from multivariable Cox proportional hazards regression models using age (in days) as the time metric and stratified by age (in years) with the adjustment for race, family history of breast cancer in first-degree relatives, combined age at first full-term pregnancy and parity variable, combined menopausal status and MHT use variable, BMI at baseline, history of smoking, alcohol intake during the past year of baseline, screening mammogram in the past 2 years of baseline, and history of a breast biopsy. ^a^Diagnosed in 1999 and afterwards. ^b^Additionally, strenuous RPA and moderate RPA mutually adjusted. ^c^RPA from high school through age 54 years or through the age at cohort entry if the woman was less than 54 years of age at baseline. ^d^RPA during the 3 years prior to baseline. Abbreviations: BMI, body mass index; CI, confidence interval; ER, estrogen receptor; HER2, human epidermal growth factor receptor 2; HR, hazard ratio; h/wk/y, hour/week/year; MHT, menopausal hormone therapy; RPA, recreational physical activity; PR, progesterone receptor; TNBC, triple negative breast cancer.
